# Aire and Fezf2, two regulators in medullary thymic epithelial cells, control autoimmune diseases by regulating TSAs: Partner or complementer?

**DOI:** 10.3389/fimmu.2022.948259

**Published:** 2022-08-30

**Authors:** Yanfei Qi, Rongchao Zhang, Yaoping Lu, Xueyang Zou, Wei Yang

**Affiliations:** Department of Immunology, College of Basic Medical Sciences, School of Public Health, Jilin University, Changchun, China

**Keywords:** Aire, Fezf2, mTECs, TSAs, autoimmune diseases

## Abstract

The expression of tissue-specific antigens (TSAs) in medullary thymic epithelial cells (mTECs) is believed to be responsible for the elimination of autoreactive T cells, a critical process in the maintenance of central immune tolerance. The transcription factor autoimmune regulator (Aire) and FEZ family zinc finger 2(Fezf2) play an essential role in driving the expression of TSAs in mTECs, while their deficiency in humans and mice causes a range of autoimmune manifestations, such as type 1 diabetes, Sjögren’s syndrome and rheumatoid arthritis. However, because of their regulatory mechanisms, the expression profile of TSAs and their relationship with special autoimmune diseases are still in dispute. In this review, we compare the roles of Aire and Fezf2 in regulating TSAs, with an emphasis on their molecular mechanisms in autoimmune diseases, which provides the foundation for devising improved diagnostic and therapeutic approaches for patients.

## 1 Introduction

In all vertebrates, T lymphocytes develop in the thymus, and T-cell receptor (TCR) diversification is a random process that is generated through somatic DNA rearrangement to provide sufficient immune coverage (1). However, this coverage comes at a cost, as it will invariably give rise to T-cell generation with the potential for autoreactivity. To protect individuals from autoimmune diseases, central and peripheral tolerance are essential to prevent the immune system from reacting against self-antigens (2). Autoreactive T cells are eliminated *via* negative selection in the thymus, a process that contributes to the establishment of central tolerance (3).

T-cell development depends on the thymic microenvironment, which is mainly supported by thymic epithelial cells (TECs). Cortical TECs (cTECs) that reside in the outer cortex region play a critical and unique role in the positive selection of T cells, while medullary TECs (mTECs) are located in the inner medulla area and are instrumental in inducing negative selection (4). In the 1980s, tissue-specific antigens (TSAs) were reported to be expressed in human and animal thymus, and these antigens were considered to exist in particular organs or tissues as cell products or cell structures (5, 6). Promiscuous expression of TSAs in mTECs is needed for the establishment of T-cell self-tolerance. In this process, mTECs with high levels of major histocompatibility complex (MHC) II molecules act as antigen-presenting cells (APCs) and can directly present TSAs to autoreactive T cells, leading to the induction of tolerance either by clonal deletion or functional inactivation (5–7). However, reduced expression and presentation of TSAs by mTECs will lead to autoreactive T cells escaping negative selection, contributing to increased susceptibility to autoimmune diseases.

Autoimmune regulator (Aire), a transcriptional regulator, is attractive because of its link to autoimmune polyendocrinopathy candidiasis ectodermal dystrophy (APECED), which is an autosomal-recessive disorder. Patients with APECED have two of the three most common clinical features, chronic mucocutaneous candidiasis (CMC), chronic hypoparathyroidism (CH), and Addison’s disease (AD) (8, 9). Moreover, several reports have identified some patients with Aire mutations who exhibit other milder autoimmune phenotypes, such as accompanying pernicious anemia (PA), vitiligo, autoimmune thyroid disease (AITD), and type 1 diabetes(T1D) (10–12). Aire is predominantly expressed in mTECs, and has been considered important for negative selection of autoreactive T cells. In addition, Aire has also been implicated in the development of mTECs, promoting antigen presentation or regulating antigen transfer from mTECs to dendritic cells (DCs) (13). More than 20 years after the discovery of Aire (14), thymic TSA expression is commonly thought to be exclusively dependent on Aire. However, the mTECs in Aire-deficient mice have been reported to have numerous TSA gene expression (15), raising the possibility that other regulators participate in the induction of self-antigens in mTECs. Another transcriptional regulator, FEZ family zinc finger 2(Fezf2), has been reported to play an indispensable role in promiscuous TSA expression in mTECs independent of Aire (16). Fezf2 deficiency in TECs leads to inflammatory cell infiltration in the lung, liver, kidney, stomach, small intestine, salivary gland, brain, and testis, but not in the pancreas or retina which are highly affected in mice lacking Aire (16). In addition, Fezf2-deficient mice have many more autoantibodies that recognize TSAs than Aire-deficient mice (16). These results suggested that the spectrum of autoimmune target tissues is possibly wider in Fezf2-deficient mice. This review will provide a comprehensive description of the role of Aire or Fezf2 in thymic TSA expression and their effects on autoimmune diseases.

## 2 Mechanisms of TSA regulation by Aire and Fezf2

Aire and Fezf2 are highly expressed in mTECs, whereas as outlined above, they directly induce the expression of numerous TSAs (**Table 1**). The latest advances in understanding how Aire and Fezf2 affect the transcription of a wide variety of genes are discussed in detail below.

**Table 1:** Examples of TSAs regulated by Aire and Fezf2.

	Gene Symbol	Gene Name	References
**Aire**	Ins2	Insulin II	(17)
	Gad65/67	Glutamate decarboxylase 65/67	(17)
	IA-2	Insulinoma-associated protein 2	(17)
	Igrp	Islet-specific glucose-6-phosphatase catalytic subunit-related protein	(17)
	Rbp3	Retinol-binding protein 3	(17)
	Spt1/2	Salivary protein 1/2	(17)
	Nalp5	NACHT leucine-rich-repeat protein 5	(17)
	Mog	Myelin-oligodendrocyte glycoprotein	(17)
	Cyp17a1/2	Cytochrome P450c 17 A1/2	(18)
	Sbp	Spermine binding protein	(19)
	Irbp	Interphotoreceptor retinoid-binding protein	(19)
	Hbby	Hemoglobin Y	(19)
	Ptgds	Prostaglandin D2 synthase	(19)
	Dcpp	Demilune cell and parotid protein	(19)
	Gpr50	G protein–coupled receptor 50	(19)
	Crabp1	Cellular retinoic acid binding protein I	(19)
**Fezf2**	Ttr	Transthyretin	(16)
	Krt10	Keratin 10	(16)
	Resp18	Regulated endocrine-specific protein 18	(16)
	Fabp9	Fatty acid binding protein 9	(16)
	Apoc3	Apolipoprotein C3	(16)
	Csrnp3	Cysteine-serine-rich nuclear protein 3	(16)
	Klk1b16	Kallikrein 1-related peptidase b16	(16)
	Maoa	Monoamine oxidase a	(16)
	Smtnl1	Smoothelin-like 1	(16)
	Calb1	Calbindin 1	(16)
	Nb1	N/A	(16)
	Timd2	T cell immunoglobulin and mucin domain containing 2	(16)
	Pck1	Phosphoenolpyruvate carboxykinase 1	(16)
	Nol4	Nucleolar protein 4	(16)
	Bbox1	Gamma-butyrobetaine dioxygenase	(16)

### 2.1 Mechanisms by which Aire regulates the expression of TSAs

A key to understanding the molecular mechanisms by which Aire regulates TSA expression is to target loci encoding TSAs. Unlike other canonical transcription factors, Aire does not bind to a specific DNA-binding domain but can act as a part of the multimolecular complex involved in gene transcription (20) (**Table 2**). According to some authors’ studies, the first plant homeodomain (PHD1) zinc finger of Aire might participate in the regulation of TSAs by binding unmethylated histone H3 lysine 4 (H3K4), which is a repressive epigenetic marker (31). However, the results of studies on H3K4 demethylase overexpression showed that global demethylation of H3K4 has no effect on TSA expression (32), suggesting that Aire binding to H3K4 is insufficient to drive TSA expression. Thus, additional mechanisms for regulating Aire-driven TSA expression are likely important.

**Table 2:** Aire-interacting partners and their functional roles.

Aire’s partner	Molecular mechanisms	Functional roles	References
ATF7ip; MBD1	Methylates CpG dinucleotides	Target specific TSA genomic loci	(21, 22)
DNA-PK; TOP2; PARP-1; FACT; Ku	Induction and religation of single- and double-strand DNA breaks, removing and reassembling histones around RNA polymerase	Transcriptional elongation	(23, 24)
P-TEFb; HnRNPL	Release of stalled RNA polymerase	Transcriptional elongation, pre-mRNA splicing	(25, 26)
CBP Sirt1	Acetylation of lysine Deacetylation of lysine	Transactivation Transcriptional activation	(27, 28) (29, 30)

ATF7ip, activating transcription factor 7-interacting protein; MBD1, methyl CpG-binding protein 1; DNA-PK, DNA-dependent protein kinase; TOP2, topoisomerase 2a; PARP-1, poly (ADP-ribose) polymerase 1; FACT, facilitates chromatin transcription; Ku, Ku80; P-TEFb, positive transcription elongation factor b; CBP, CREB-binding protein; Sirt1, Sirtuin-1.

It should be mentioned that additional marks of a repressive chromatin state were linked with Aire. Aire targets activating ATF7ip-MBD1complex, which is associated with the histone methyltransferase ESET-SETDB1 targeting it to methylated CpG dinucleotides that are rich in inactive gene promoters. Furthermore, mTECs from MBD1-deficient mice showed decreased expression of Aire-dependent TSAs (21). Athymic nude mice that received MBD1-deficient thymus develop autoimmune diseases, indicating that thymic MBD1 may provide a protective effect against the development of autoimmune disease. Of note, the combined absence of both MBD1 and ATF7ip through shRNA *in vitro* led to a significant absence of Aire-dependent TSAs. These reports imply that Aire-regulated TSA expression requires these two proteins.

More detailed studies on the TSAs in mTECs from SCID mice with DNA-PK mutations versus their WT counterparts have provided new evidence that DNA-PK appeared to be critical for Aire to promote the expression of TSAs (23, 24). DNA-PK is identified as a nuclear kinase responsible for the repair of DNA double‐strand breaks (DSBs) and DNA replication. DSBs trigger histone H2AX phosphorylation at Ser-139 (γH2AX), and DNA-PK cooperates with Aire binding to γH2AX (24). Thus, DNA-PK may direct Aire to TSAs poised for transcription, as DSBs that result in DNA damage are associated with transcription initiation, which may be part of the cellular mechanism by which Aire regulates TSA expression in mTECs.

Unlike typical transcription factors, Aire promotes TSA expression through RNA elongation rather than gene transcription initiation. RNA transcripts fail to elongate in Aire-deficient mTECs, suggesting a strong induction of Aire on RNA elongation. Previous work showed that P-TEFb, as an Aire partner, favors the release of stalled RNA polymerase to elongate RNA transcripts (25). Another report identified HnRNPL as another Aire partner, which was coimmunoprecipitated with Aire and the P-TEFb components CDK9 and HEXIM1, whose deletion in mTECs inhibited the transcription of the P-TEFb components CCNT2 and CDK9, suggesting a role for HnRNPL in RNA elongation (26). 7SK snRNA is a noncoding RNA that can interact with Aire and whose presence is critical for the regulation of P-TEFb activity (26). It is now clear that the loss of HnRNPL reduces this interaction and provides a detailed mechanism of how HnRNPL promotes the interaction of Aire with P-TEFb. In addition, Peterlin et al. found that Aire can regulate precursor messenger RNA (pre-mRNA) splicing *via* P-TEFb or other factors (26, 33). Aire upregulates pre-mRNA splicing of heterologous minigenes through P-TEF-b and associates with multiple interacting partners (e.g., EFTUD2, SNRPB, SRSF1) (33). Overall, Aire-regulated expression of TSAs is linked to RNA elongation and pre-mRNA splicing instead of transcription initiation.

The integrity of the caspase recruitment domain (CARD) and SAND domains of Aire with transactivation ability is critical for Aire-dependent TSA expression. Aire expression is required for CBP translocation from the cytoplasm to the nucleus, which is an activity driven by a CARD-dependent interaction that accelerates the transactivation potential of Aire (27). RANKL, as a member of the tumor necrosis factor family, has been reported to promote Aire expression in mTECs by activating NF-κB2, and accumulation of Aire and CBP in anti-RANK-stimulated nuclei may drive the focal induction of TSAs (34). CBP as acetyltransferase proteins can act directly upon Aire (28). At the same time, Aire also interacts with Sirt1, a protein which plays a critical role in deacetylation of lysine on the Aire protein and regulation of Aire-dependent TSA expression (29, 30). The binding of Aire to DNA has been a source of intense study. Aire has been shown to bind specific DNA sequences *in vitro* (35). Several studies have identified the three-dimensional structure of the SAND domain in the Sp100b protein as well as additional related proteins, such as AIRE1, NucP41/P75 and DEAF-1. SAND domains may mediate DNA binding of these proteins in the context of transcriptional regulation (36, 37). Thus, it may be possible that the SAND structure of Aire participates in regulating TSA expression in mTECs by binding to its specific target DNA sequences.

### 2.2 Mechanisms of Fezf2 regulating the expression of TSAs

In 2020, Tomofuji and colleagues used RNA-Seq to examine downregulated genes in mTECs from mice lacking Fezf2 or Aire. They identified 640 genes and 1,553 genes regulated by Fezf2 and Aire, respectively (38). Among them, 123 genes that were dependent on both factors were identified (38). Bioinformatics analysis further showed that more than 60% of the TSAs in mTECs are regulated by Aire and/or Fezf2. Of note, Fezf2 is highly expressed in mTEC^hi^ cells. Consistently, mTEC^hi^ cells had higher levels of Fezf2-dependent genes than mTEC^lo^ cells (16). The repressed genes in Fezf2 ^-/-^ mTECs are reported to be associated with TSAs in autoimmune diseases or tumors. Takayanagi et al. performed a genome-wide analysis of mTECs from Fezf2-deficient mice and selected genes with more than a fourfold reduction in gene expression compared to the wild-type control. It was found that 16 Fezf2-dependent genes were TSAs, among which the expression of Krt10, Resp18, Fabp9, Maoa, and Timd2 was not regulated by Aire, that is, Aire-independent TSAs (16). Overall, Fezf2 is shown to regulate a unique subset of TSA genes independent of Aire.

Is the regulation of TSA expression by Fezf2 independent of Aire? The applications of affinity propagation clustering revealed coherent patterns of genes whose expression is gene-by-gene correlated for Aire-regulated genes (39), suggesting a high degree of coexpression at the single-cell level. However, such pattern was not observed in Fezf2-regulated genes (38). Gene expression regulated by Fezf2 is different from that of Aire- regulated genes, which is suggested by the observation that Fezf2- regulated gene expression exhibits broad patterns in mTECs rather than mosaic patterns (38). A study found that Fezf2 binds to the promoters of multiple protein-coding genes in cortical progenitor cells (40). Subsequent research applied ChIP-seq analysis and found that Fezf2 was directly bound to the promoter of Fezf2-dependent TSAs, such as Mbp, Gad1, Col2a1, and Muc1, and other autoantigens but not to the promoter of Aire-dependent TSAs in mTECs (16). In addition, data from luciferase reporter assays revealed that Fezf2 regulates the expression of additional TSAs, such as Maoa, Calb1, and Nol4 (16). Thus, these results suggest a direct role of Fezf2 on TSA expression by binding to the promoter region, which is different from the Aire-dependent manner. Furthermore, the Irla group found higher levels of trimethylated lysine 4 (an active histone mark) of histone 3 (H3K4me3) at the transcription start sites (TSSs) of Fezf2-dependent genes than on Aire-dependent genes, while the trimethylated lysine 27 (a transcriptional repressor) mark of histone 3 (H3K27me3) was more enriched on Aire-dependent gene TSS than on Fezf2-dependent genes (41). At the same time, TSSs of Fezf2-dependent genes were also more enriched for other active transcription markers, such as H3K4ac and H3K9ac, in comparison with their enrichment to Aire-dependent genes (38). Here, determination of transposase-accessible chromatin using sequencing (ATAC-Seq) showed higher signal intensity around the TSS of Fezf2-regulated genes (38). Thus, these results suggest that Fezf2-dependent TSA histone modifications are distinctly different from Aire-dependent TSAs.

## 3 TSA expression in mTECs regulated by Aire and Fezf2 is involved in autoimmune diseases

It is well-established that mutation of Aire results in multiple organ-specific autoimmune diseases, and mice with Fezf2 deficiency in mTECs also develop a severe autoimmune phenotype, which is thought to result from a failure of the given thymic TSA expression under the control of Aire or Fezf2. A clear link between selective single thymic TSAs and spontaneous organ-specific autoimmune disease in the mouse model has been well-reported. Here, we review some of the recent progress in our understanding of individual TSAs whose thymic expression is regulated by Aire or Fezf2, which are critical for specific autoimmune diseases.

### 3.1 TSA expression in mTECs regulated by Aire is involved in multiple autoimmune diseases

#### 3.1.1 Thymic Irbp prevents autoimmune uveitis *via* an Aire-dependent mechanism

Approximately one-third of Aire-deficient mice with B6 genetic background develop spontaneous autoimmune uveitis between the ages of 10 and 20 weeks, the severity of which increases with age and is characterized by lymphocytic infiltration and retinal autoantibodies in the photoreception layer (42, 43). The development of the uveitic process in mice lacking Aire is directly dependent on the response to the retina-specific antigen Irbp (42, 43), whose expression within the thymus maps to mTECs, where it is induced in an Aire-dependent manner, and Irbp -deficient thymus transplantation into athymic nude mice is sufficient to induce posterior uveitis (42, 43). Taniguchia et al. investigated Aire-mediated thymic deletion of Irbp -specific T cells using a method to detect rare antigen-specific populations in polyclonal T-cell repositories (43). The function of Aire on thymic deletion of autoreactive T cells specific for a peptide epitope of amino acids 271–290 of Irbp (P2) has been confirmed by the observation that P2-specific T cells were barely detectable in Aire-sufficient mice following immunization with full-length P2 emulsified in complete Freund’s adjuvant (CFA) (43). Interestingly, in the same study, in contrast to the loss of P2-specific T cells, mice with P7-CFA immunization showed greatly expanded T cells specific for the peptide epitope of amino acids 771–790 of Irbp (P7), suggesting that P7-specific autoreactive T cells escaped Aire-mediated deletion in the thymus. Of note, a pathogenic-specific P2 epitope is critical in driving the progression of uveitis because it can induce uveitis in an adoptive transfer mouse model, and young Aire-deficient mice challenged with P2 peptides can develop uveitis at a time point before spontaneous disease (43).

#### 3.1.2 Thymic Obp1a prevents Sjögren’s syndrome *via* an Aire-dependent mechanism

Sjögren’s syndrome (SS) is an autoimmune disease characterized by lymphocytic infiltration and immune-mediated destruction of the lacrimal and salivary glands. Transplantation of Aire-deficient matrix into nude mice results in retinal and salivary autoimmunity, indicating that Aire deficiency may trigger SS (44). Thus, Aire-deficient mice are likely to serve as a new tool for studying this autoimmune disease. Using an unbiased biochemical approach, Anderson et al. found that a novel autoantigen in lacrimal glands is identified as odorant binding protein 1a (Obp1a), which is a putative pheromone transporter that is part of the lipocalin family. This type of TSA is promiscuously expressed in the lacrimal gland and the vomeronasal organ, and some expression was also observed in the thymus (44). A study with Aire-regulated TSAs suggests that male mTECs have higher Obp1a than female mTECs (45). An Obp1a -GST fusion protein produced in E. coli was used to detect autoantibodies against Obp1a in the serum of Aire-deficient mice (44). The obtained results showed that the Obp1a autoantibodies in Aire-deficient mice were higher than those in age-matched controls. At the same time, infiltration of CD4^+^, CD8^+^ T cells and IgD^+^ B cells was detected in the lacrimal glands of mice lacking Aire, while no infiltration of these immune cells was detected in tissues from control animals. Further research found that three-quarters of Aire-deficient mice had specific T cells against Obp1a in the lacrimal gland, indicating that the increase in autoantibodies and immune infiltration in the lacrimal gland caused by Aire deficiency may be achieved by mediating the expression of Obp1a (44). Furthermore, cervical lymph node cells (LNCs) from Aire-deficient mice were stimulated with Obp1a *in vitro* and then transplanted into age- and sex-matched scid immunodeficient recipients with severe lacrimal duct disease (44). Taken together, Obp1a expression in mTECs may be a possible mechanism of spontaneous SS in the Aire-deficient mouse model.

#### 3.1.3 Thymic insulin prevents T1D *via* an Aire-dependent mechanism

APECED is caused by mutations in Aire, with 18% of cases including T1D (46). It is well known that T1D is a metabolic disorder syndrome defined by the absolute loss of insulin secretion due to autoimmune destruction of beta cells in the pancreas. Insulin is thought to be a key TSA driving T1D development; it is promiscuously expressed in the thymus and is critical in the maintenance of central tolerance, while dysregulation of insulin in mTECs leads to T1D development in mice and humans (47, 48). Thymic insulin expression is regulated by Aire, it must first interact and bind with the insulin-variable number of tandem repeats (INS-VNTR), which contain a unique polymorphic tandem repeat sequence with the insulin basal promoter (49, 50). Lan et al. found that G228W and R257X mutations in SAND, C311fsX376 and L397fsX478 mutations in PHD1 and R433fsX502 mutations in PHD2 of Aire resulted in inhibition of INS-VNTR transcription, which may contribute to inactivity of the insulin in mTECs and subsequently induce insulin resistance or T1D (49). In addition, the expression of the pancreatic transcription factor Pdx1 in the thymus is also dependent on Aire, and this molecule is required for insulin expression in islets (51). Thus, Dooley et al. speculated whether Aire could indirectly regulate islet-specific genes by inducing thymic Pdx1 expression, which then directly activates the expression of its downstream TSAs, such as insulin (52). They used a mouse model with Pdx1 deletion to detect the expression of insulin in the thymus. The obtained results showed that the transcription of insulin in mTECs was not affected by Pdx1 deletion, suggesting that Aire is regulated independently of Pdx1 expression in the thymus, further supporting a direct transcriptional activation effect of Aire on insulin in the thymus.

#### 3.1.4 Thymic Chrna1 prevents myasthenia gravis *via* an Aire-dependent mechanism

Myasthenia gravis (MG) is a chronic autoimmune disease triggered by antibodies against the acetylcholine receptor (AChR) of skeletal muscle at the neuromuscular junction, resulting in muscle weakness and fatiguability of skeletal muscles. Aire-deficient mice express lower levels of the AChR than wild-type (WT) mice and present increased susceptibility to induction of experimental autoimmune MG (53), suggesting that Aire plays a critical role in the immunopathogenesis of MS. A possible mechanism of Aire in MG development was suggested by the observation that mTECs from Aire-deficient mice express significantly decreased acetylcholine receptor subunit alpha-1 (Chrna1), a gene coding for AChR, which is also a main target of pathogenic autoantibodies in MS (54, 55). The regulation of Aire on Chrna1 has also been confirmed in human mTECs (56). Of note, there were no significant differences in the expression of Chrna1 in the muscle of Aire- deficient mice and WT mice (55), further suggesting that the reason for the susceptibility of Aire- deficient mice to MG may be associated with Chrna1 expression in the thymus rather than in their muscle.

### 3.2 TSA expression in mTECs regulated by Fezf2 is involved in multiple autoimmune diseases

#### 3.2.1 Thymic Ttr prevents rheumatoid arthritis *via* an Fezf2-dependent mechanism

Rheumatoid arthritis (RA) is a chronic, complex autoimmune inflammatory rheumatic disease that primarily causes joint deformation and inflammation of surrounding tissues and affects approximately 0.5-1% of the population (57). In addition to clinical symptoms, autoantibody reactivity against self-antigens has gradually become the diagnostic criterion for RA. In 2014, the application of proteomic techniques observed that 71% of RA patients had significantly upregulated transthyretin (Ttr) in plasma (58). Of note, Ttr expression increases with the severity of RA (58). Therefore, Ttr can be used as a serum diagnostic marker along with other biochemical parameters and clinical symptoms for RA screening and diagnosis. Furthermore, the levels of autoantibodies against Ttr were significantly upregulated in RA patients (58), further illustrating the important role of Ttr in the pathogenesis of RA. A report found that TEC-specific Fezf2-deficient (Foxn1Cre^+^Fezf2^fl/–^) mice have autoantibodies that recognize joint tissue antigens, and approximately 30% of these mice died at 12 weeks of age (16). This means that Fezf2 deficiency can cause severe autoimmune symptoms. Of note, the sera obtained from Foxn1^-^Cre^+^Fezf2^fl/–^ mice showed a marked increase in autoantibodies recognizing Fezf2-dependent TSAs, including Ttr (16). These results suggest that Fezf2 plays a critical role in mTECs to ensure immune tolerance to Ttr and potentially inhibit the development of autoimmune diseases such as RA.

#### 3.2.2 Thymic Resp18 may prevent Parkinson’s disease *via* an Fezf2-dependent mechanism

Parkinson’s disease is a progressive neurological disorder that primarily affects dopamine-producing neurons in the substantia nigra of the brain and usually causes stiffness or slow movement. Several studies have suggested that Parkinson’s disease is partly an autoimmune disease (59, 60). The striatum and substantia nigra, two regions affected by Parkinson’s disease, abundantly express Resp18, a molecule that was recently shown to have an important role in Parkinson’s disease (61, 62). Continuous administration of the dopaminergic toxin 1-methyl-4-phenyl-1,2,3,6-tetrahydropyridine (MPTP) for 5-6 days can be used to induce wild-type mice to generate an animal model of Parkinson’s disease with symptoms such as hyperactivity, decreased motor coordination and loss of dopaminergic neurons (62). However, the application of MPTP had no effect on the activity and motor coordination of Resp18-deficient mice, and neuronal damage was significantly lower than that in wild-type mice (62). Resp18 deficiency may prevent the development of Parkinson’s disease by blocking MPTP-induced microglial activation and preserving dopamine neurons. Fezf2 is sufficient to increase dopaminergic neurons, and its defective expression may contribute to the progression of disease phenotypes such as Parkinson’s disease (63). Takayanagi et al. performed a genome-wide analysis of mRNA expressed in mTECs isolated from Fezf2-deficient mice, and the expression of Resp18, an Fezf2-dependent TSA, was significantly reduced in Fezf2-deficient mTECs (16). However, there is no evidence to prove that thymic Resp18 prevents Parkinson’s disease *via* an Fezf2-dependent mechanism, it will be important in future studies to investigate the possibility that Fezf2 is involved in the progression of Parkinson’s disease by affecting the expression of Resp18 in mTECs.

#### 3.2.3 Thymic Aqp8 prevents Sjögren’s syndrome *via* an Fezf2-dependent mechanism

Aquaporin (Aqp) is expressed in the salivary glands, and is mostly localized in the cytoplasm of excretory ducts. Despite the presence of several antibodies against Aqps, the highest frequency of antibodies to Aqp8 has been found in the sera of patients with SS, and this type of antibody is associate with the severity of xeropthalmia (64), indicating that Aqp8 is the main actor involved in the immune mechanisms in SS. Takayanagi et al. found that Aqp8 expression is dependent on Fezf2 (38). The fact that the deficiency of Fezf2 in TECs is significantly correlated with inflammatory cell infiltration in the salivary gland supports the view that Fezf2 may have a key role in SS (16). These results suggest that Fezf2 is involved in the progression of SS by affecting the expression of Aqp8.

## 4 Conclusion and perspectives

**Figure 1:**
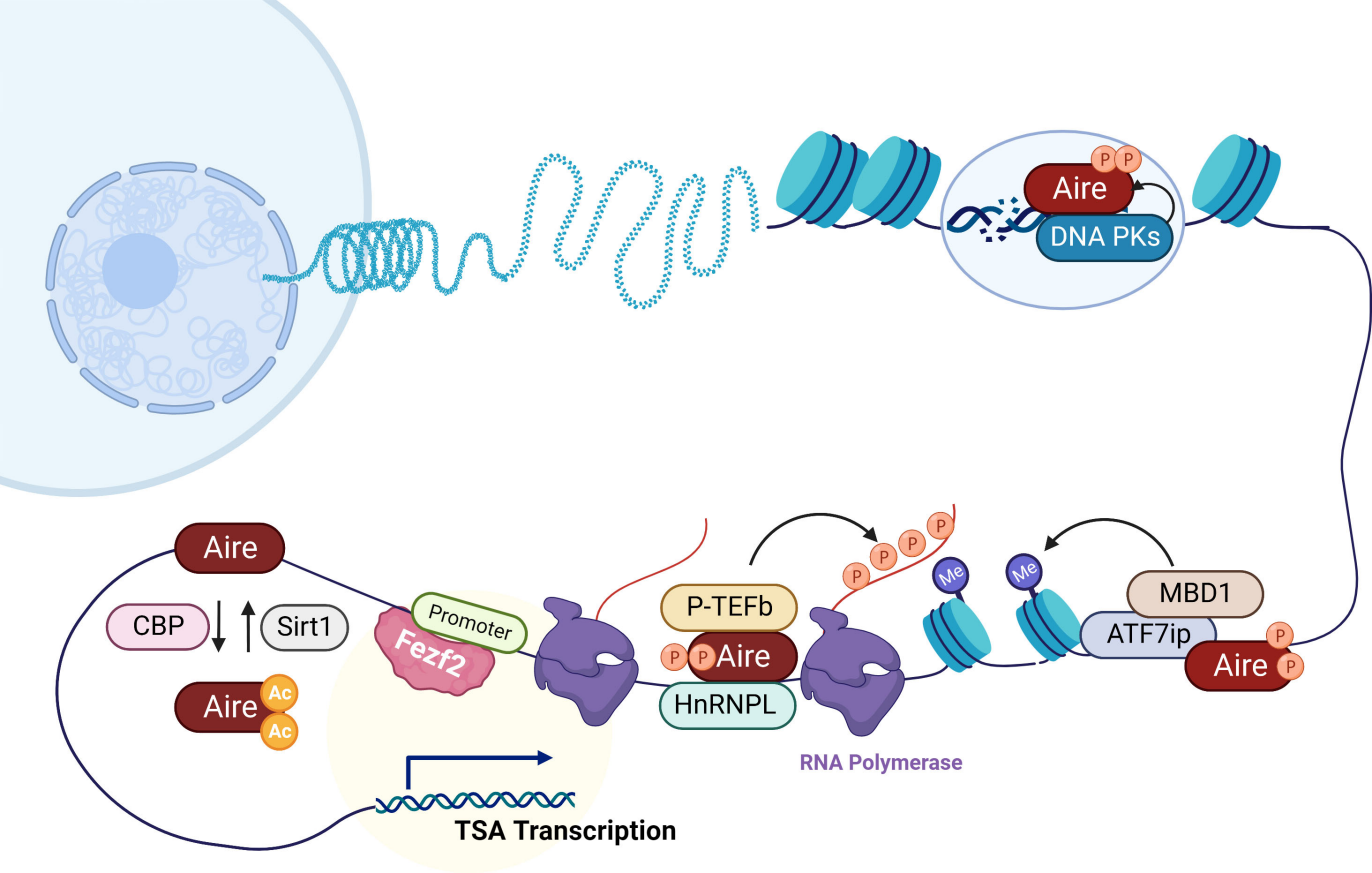
Mechanisms of Aire and Fezf2 in the transcription regulation of TSAs in mTECs. Aire preferentially targets activating ATF7ip-MBD1 which interacts with methylated DNA, causing TSA expression in mTECs. In addition, here is shown Aire’s interaction with DNA-PK which is responsible for the repair of DSBs that are associated with transcription initiation. Aire also recruits P-TEFb and HnRNPL to favor the release of stalled RNA polymerase for elongation of Aire-dependent TSAs. CBP and Sirt1 respectively acetylates and deacetylates Aire that regulate Aire-regulated TSA expression. Unlike Aire, Fezf2 regulates TSA expression in mTECs by binding to the promoters.

**Figure 2:**
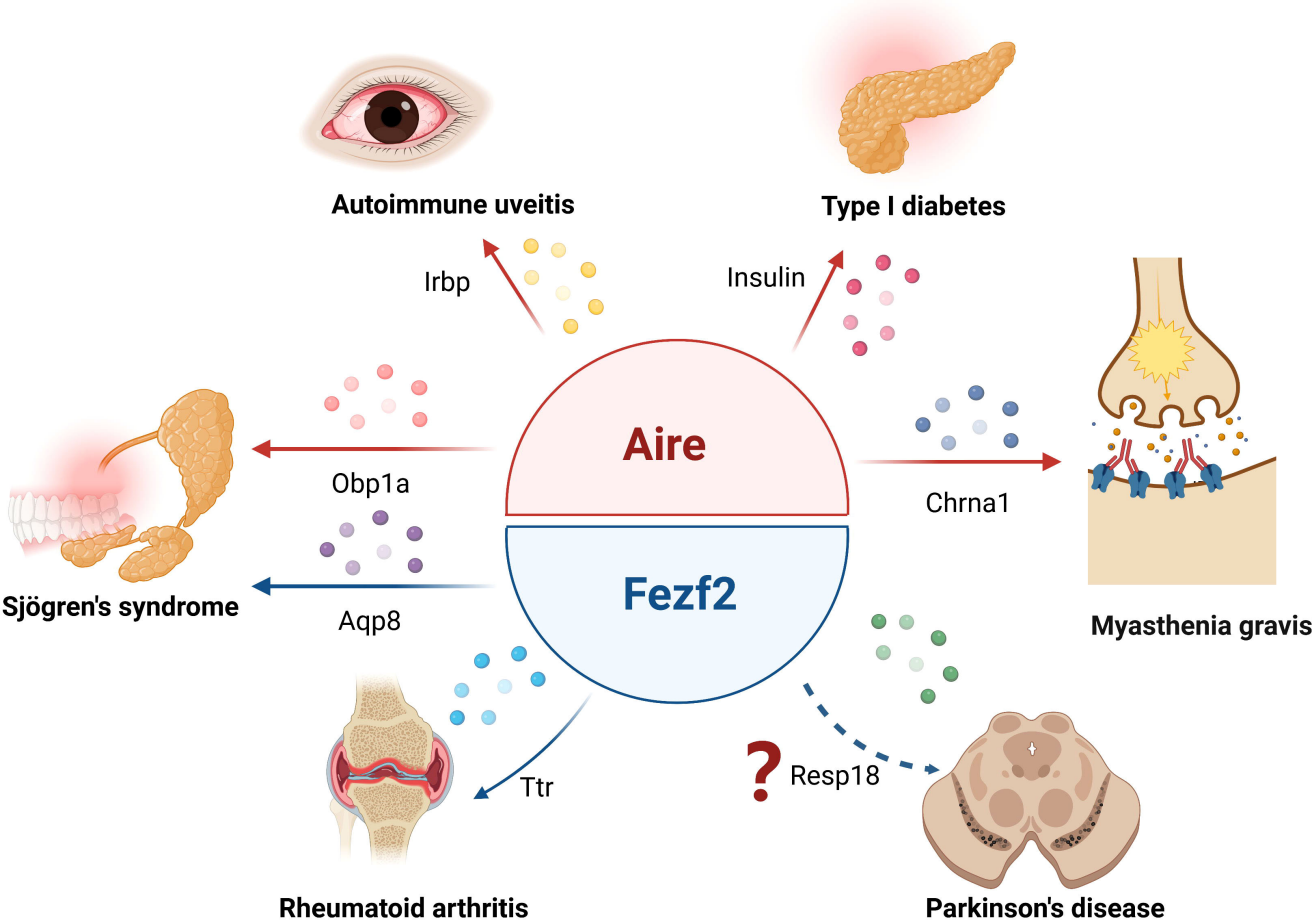
Aire and Fezf2 in mTECs control autoimmune diseases by regulation of TSAs. Aire respectively regulates the expression of Irbp, Obp1a, insulin or Chrna1 in mTECs, participating in the development of autoimmune uveitis, Sjögren’s syndrome, type 1 diabetes or myasthenia gravis. Fezf2- dependent Ttr or Aqp8 expression in mTECs is involved in the regulation of rheumatoid arthritis or Sjögren’s syndrome phenotype in mice, Fezf2 may prevent Parkinson’s disease by regulating the expression of Resp18 in mTECs.

TECs, especially mTECs, can promiscuously express tissue-specific autoantigens, which are important for the negative selection of autoreactive T cells, thus participating in the establishment of central tolerance. It has long been believed that thymic TSA expression is completely dependent on Aire. In the last decades, a growing body of research points to the regulation of Fezf2 on TSA expression in mTECs in an Aire-independent manner. Of note, Fezf2 and Aire mediate TSA expression with distinct mechanisms (**Figure 1**). Aire has no specific DNA binding domain or transcriptional activity, and it interacts with several nuclear factors, such as the ATF7ip-MBD1 protein complex and DNA-PK, or releases stalled RNA polymerase to promote TSA gene transcription. In contrast, Fezf2 directly binds to the TSA promoter to induce gene expression. Thus, two independent molecular mechanisms lead to different Fezf2- and Aire-dependent TSA expression patterns, which cause Fezf2 and Aire respectively participate in diverse autoimmune diseases (**Figure 2**). For example, Aire can participate in the occurrence and development of autoimmune uveitis, SS, T1D or MG by regulating the expression of Irbp, Obp1a, insulin or Chrna1 in mTECs, respectively. The expression of Ttr or Aqp8, regulated by Fezf2 in mTECs, is involved in RA or SS in mice. Fezf2 may prevent Parkinson’s disease by regulating the expression of Resp18 in mTECs.

In addition to the autoimmune diseases mentioned above, Aire may also participate in the occurrence of other autoimmune diseases by regulating the expression of additional TSAs. The antibodies in the sera of Aire-deficient mice with experimental autoimmune prostatitis (Eap) were found to react with prostatic autoantigens, including Eapa1 and Eapa2, but RT–qPCR results showed that Eapa1 was undetectable in TECs, while Aire deletion had no effect on the expression of Eapa2 on TECs, indicating that Aire is not involved in this autoimmune disease by regulating Eap-related TSAs on TECs (65). In addition, several studies have found that Aire deletion markedly inhibited the expression of TSAs in mTECs, including Fabp2, Cyp1a2 and Ica69 (65). Whether this mechanism is involved in the development of autoantigen-related autoimmune diseases remains to be determined. Given the complexity of Fezf2 function, there are still some issues to be resolved. First, there is no genetic or pathological evidence directly linking Fezf2 defects to specific autoimmune diseases, although mice lacking Fezf2 in mTECs develop a severe autoimmune phenotype and produce autoantibodies and inflammatory cell infiltration. Second, in addition to directly binding to the TSA promoter region to induce gene expression, we wondered whether Fezf2 can also indirectly regulate the expression of TSAs in mTECs through other pathways, and what is the specific mechanism? Third, Fezf2-dependent genes include certain TSAs associated with autoimmune diseases: Amy2a is associated with autoimmune pancreatitis and fulminant T1D, and CD177 has been reported to be associated with autoimmune neutropenia. More work will be needed to determine whether the development of related autoimmune diseases can be regulated by Fezf2- dependent TSAs in mTECs.

In recent years, multiple roles for Aire beyond its functions on TSAs have been described. Tregs can suppress autoreactive cells and protect a defined set of tissues from autoimmune attack, thereby maintaining homeostasis and self-tolerance. Previous studies have shown that Aire-deficient mice have a partial reduction in Treg number (66) or the suppressive signature of recirculating Tregs from the periphery (67). However, whether these Tregs are specific for Aire- dependent TSAs is not currently known. The observations by Takahama et al. suggest that Aire may indirectly promote Treg development by regulating mTEC expression of XCL1, which is a chemokine that affects the migration of DCs into the thymus (68). Similar results were observed in TEC-specific Fezf2-deficient mice, and the percentages of Tregs were reduced in the lymph nodes (16), suggesting a role for Fezf2 in regulating Treg development. Thus, Aire and Fezf2 are likely to have a broad effect on immune tolerance through multiple effects. However, there is still much that needs to be learned about the mechanism by which these two transcription factors regulate Treg development and to what extent these effects contribute to the autoimmune phenotype observed in Aire- or Fezf2-deficient mice and APECED patients.

Above studies have expanded our knowledge of the role of Aire and Fezf2 and will help to refine any strategy aimed at restoring, promoting, or strengthening the mechanisms of central and peripheral self-tolerance. Although only a small portion of TSAs are regulated by Aire and Fezf2 at the same time, it is unclear whether these two transcription factors play a synergistic or independent role. On the other hand, the use of modern techniques, such as single-cell sequencing, may provide more information about the regulation of TSAs by Aire or Fezf2. The transcription factors regulating TSA expression may be a large family. PR domain zinc finger protein 1 (Prdm1) is another transcription factor expressed in mTECs that is reported to regulate TSA expression independently of Aire. Importantly, epithelial deletion of Prdm1 results in autoimmune diseases, such as systemic lupus erythematosus (69). It is conceivable that more members may be found in the future.

## Author contributions

WY and XZ conceptualized the study. YQ drafted the manuscript. RZ and YL conducted the literature review. All authors contributed to the article and approved the submitted version.

## Funding

This review was supported by grants from the National Natural Science Foundation of China (no. 81671548) and project of Health Commission of Jilin Provincial (No. 2021JL035).

## Conflict of interest

The authors declare that the research was conducted in the absence of any commercial or financial relationships that could be construed as a potential conflict of interest.

## Publisher’s note

All claims expressed in this article are solely those of the authors and do not necessarily represent those of their affiliated organizations, or those of the publisher, the editors and the reviewers. Any product that may be evaluated in this article, or claim that may be made by its manufacturer, is not guaranteed or endorsed by the publisher.
